# Social Distancing and Economic Crisis During COVID-19 Pandemic Reduced Cancer Control in Latin America and Will Result in Increased Late-Stage Diagnoses and Expense

**DOI:** 10.1200/GO.21.00016

**Published:** 2021-05-17

**Authors:** Tabaré Vázquez Rosas, Eduardo Cazap, Lucía Delgado, Julia Ismael, Suyapa Bejarano, Carlos Castro, Hugo Castro, Bettina Müller, Francisco Gutiérrez-Delgado, Luiz Antonio Santini, Carlos Vallejos Sologuren

**Affiliations:** ^1^ Radiation Oncology, University of Uruguay, Montevideo, Uruguay; ^2^ Latin-American and Caribbean Society of Medical Oncology—SLACOM, Buenos Aires, Argentina; ^3^ Clinical Oncology, Universidad de la República, Former Director of the National Cancer Control Program, Montevideo, Uruguay; ^4^ Clinical Oncology, Former Director of National Cancer Institute, Buenos Aires, Argentina; ^5^ Liga Contra el Cáncer, San Pedro Sula, Honduras; ^6^ Liga Colombiana Contra el Cáncer, Bogotá, Colombia; ^7^ Medical Oncology, Guatemala City, Guatemala; ^8^ Instituto Nacional del Cáncer, Santiago, Chile; ^9^ Latin American School of Oncology (ELO), Mexico City, Mexico; ^10^ Oswaldo Cruz Foundation (Fiocruz), Former Director of National Cancer Institute, Rio de Janeiro, Brazil; ^11^ ONCOSALUD-AUNA, Lima, Peru; † Deceased.

## Abstract

Since December 2019, the world has been mired in an infectious pandemic that has displaced other health priorities for 21st century populations. Concerned about this situation, Latin American experts on cancer decided to evaluate the impact of the pandemic on cancer control in the region. The analysis was based on information obtained from public sources and scientific publications and included the characteristics of the health care and cancer control prior to the pandemic, the COVID-19 pandemic and measures implemented by the governments of the region, and the regional impact of the pandemic on cancer control together with the costs of cancer care and possible impact of the pandemic on cancer expense. We compared 2019 and 2020 data corresponding to the period March 16-June 30 and found a significant reduction in the number of first-time visits to oncology services (variable depending on the country between –28% and –38%) and a corresponding reduction in pathology (between –6% and –50%), cancer surgery (between –28% and –70%), and chemotherapy (between –2% and –54%). Furthermore, a significant reduction in cancer screening tests was found (PAP smear test studies: between –46% and –100%, mammography: between –32% and –100%, and fecal occult blood test: –73%). If this situation becomes a trend, the health and economic impact will be compounded in the postpandemic period, with an overload of demand on health services to ensure diagnostic tests and consequent treatments. On the basis of this information, a set of prevention and mitigation measures to be immediately implemented and also actions to progressively strengthen health systems are proposed.

## INTRODUCTION

Since December 2019, the world has been mired in an infectious pandemic that has displaced other health priorities for 21st century populations. This has been detrimental to other health problems, including noncommunicable diseases, which are recognized as the leading cause of preventable illness and premature death.

**CONTEXT**

**Key Objective**
What is the magnitude of the impact of social distancing and the economic crisis during the COVID-19 pandemic on cancer control in Latin America?
**Knowledge Generated**
The pandemic plunged the world into a global crisis that affects the prevention, diagnosis, and treatment of cancer. This will result in increased cancer late diagnosis and mortality with a higher overall economic cost. Latin America was at that time in different stages of cancer control, albeit heterogeneously. This work provides unpublished evidence on the impact of the pandemic in the region on the basis of the comparison of the prepandemic and during the time of COVID-19 in Latin American countries. We found a significant reduction across the continuum of cancer care and a notable reduction in screening studies (when they were not discontinued). On the basis of these results, we propose prevention and mitigation measures that take into account the characteristics of our populations and healthcare systems.
**Relevance**
This information could become a fundamental tool for the strategies of policy makers to design efficient policies in cancer control.

According to the WHO, noncommunicable diseases are responsible for three of every four deaths in the world. Cancer is the second most frequent, after cardiovascular diseases.^1^

Likewise, in Latin America and the Caribbean, cancer ranks second as a cause of death, with 672,758 deaths from cancer recorded in 2018. During 2018, the incidence of new cases rose to 1,412,732. Looking forward, because of the region's aging population and changes in lifestyle, the incidence will increase significantly in coming years.^2^

Globally, cancer is one of the leading health challenges. In addition to its importance as a cause of death and suffering, there are increasing incidence rates and rising costs of health care in oncology.^3^

Worldwide, almost 70% of cancer deaths occur in countries ranking as medium or low on the Human Development Index. Poverty, including less access to education and health care, exposes residents to greater risk of developing and dying from cancer. According to WHO, in low-income countries, < 30% of patients diagnosed with cancer have access to treatment and in high-income countries, more than 90% of patients diagnosed with cancer have access to treatment.^4^ At the same time, the development of cancer affects productivity and family income. This, added to the high costs of treatment, impoverishes families and is an obstacle to the development of countries, helping to widen the gap between the richest countries and those with the lowest incomes.

Published studies on COVID-19 and cancer show that patients with cancer with active disease have a higher risk of serious complications and mortality from COVID-19 than the general population, particularly those with lung neoplastic involvement, myeloid suppressive treatments, advanced age, compromise of their functional status, and/or comorbidities.^5-9^ Added to the risk of dying from severe complications is the risk resulting from the overflow of the health system in the context of a severe outbreak.

Furthermore, the immediate demands of the COVID-19 pandemic have required health systems to focus on containment strategies to minimize mortality. The prioritization of COVID-19 and implementation of physical distancing as an intervention strategy has impaired cancer health providers' functioning specifically by postponing cancer screening, in-person consultations, and control tests, as well as limiting treatments that might result in significant risk of infectious complications or require critical care. The impact of public health measures to contain the pandemic is interwoven with the changes in habits, behaviors, and healthy behaviors and the consequences of the economic crisis, resulting in an increase in poverty and challenges for patients to access cancer screening and treatment from clinicians in a timely manner.^10,11^

Three recent studies from the United Kingdom estimated the possible increase in cancer mortality as a consequence of the pandemic.^12-14^ Lai et al reported a 76% reduction in the number of patients referred for a possible cancer diagnosis and a 60% reduction in chemotherapy treatments compared with pre–COVID-19 levels. The study concludes that in the 12 months following the pandemic, mortality could increase by 20%-30% in patients with cancer.^12^ Sud et al used observational studies to generate daily risk rates for cancer progression and applied them to survival by age and disease stage. They estimated that a delay per patient of 3/6 months would cause the attributable death of 4,755/10,760 of the 80,406 long-term surviving patients of the total number of patients operated on annually in England for the most frequent invasive cancers in adults.^13^ On the other hand, Maringe et al analyzed the possible increase in mortality 5 years after diagnosis for breast (7.9%-9.6%), colorectal (15.3%-16.6%), esophagus (5.8%-6.0%), and lung (4.8%-5.3%) cancers with respect to the prepandemic period. As shown, the greatest increase would be observed in relation to colorectal cancer.^14^

In addition to the negative impact on survival, diagnosis in later stages will determine a significant increase in cancer care costs compared with prepandemic period. This is important given the significant economic impact of cancer. Indeed, the total annual economic cost of cancer in 2010 was estimated at approximately 1.16 trillion in US dollars (USD) and is increasing.^3^ Although it affects all countries, it especially affects those with the lowest incomes.

In this regard, a recent Australian study estimated the excess mortality and also the economic impact resulting in diagnostic and treatment delays for four cancers (breast, lung, colorectal, and melanoma) because of the COVID-19 pandemic.^15^ To do this, they used a stage change model of the disease.^16^ Considering a 3/6-month delay in diagnosis and initiation of treatment, this study predicts almost 90/350 excess deaths in Australia and 12/46 million Australian dollars (8.6/33 million USD) in healthcare costs for 5 years for patients diagnosed with these four cancers in 2020. The authors emphasize that more accurate data on disease stage during and after the COVID-19 pandemic are essential to obtain more reliable estimates.

Concerned about this situation, a group of Latin American experts on cancer, under the leadership of Dr Tabaré Vázquez, and on the basis of experience from Uruguay (Document Proposals for a National Strategic Plan in response to the impact of the COVID-19 pandemic. Uruguay, May 6, 2020), decided in May 2020 to carry out a project with the aim to evaluate the impact of the pandemic on cancer control in the region.

The project's strategy consisted of convening health leaders from Argentina, Brazil, Chile, Colombia, Honduras, Mexico, Nicaragua, Peru, and Uruguay to operationalize country-specific technical teams that would address clinical, economic, health system, and public policy issues for cancer control in what would become this Latin American Report.

Information from countries reported in public sources and scientific publications on health systems, cancer control prior to the pandemic, the characteristics of the COVID-19 pandemic, the measures implemented by governments, and the impact of the pandemic on cancer care and expense in the short and medium term was collected and analyzed. Concrete proposals were detailed to prevent and mitigate the negative impact of the pandemic on cancer control and expense.

## CHARACTERISTICS OF THE HEALTH CARE AND CANCER CONTROL PRIOR TO THE PANDEMIC

The percentage of the GNP for health in the region ranges from 6.61% to 2.08%. Argentina (6.61%) and Uruguay (6.58%) are the countries with the largest investment in health care, whereas Mexico (2.77%) and Guatemala (2.08%) are the countries with the lowest investment levels.^17^

Funding for the healthcare systems in Latin America comes mainly from government funds, through taxes, and to a lower extent from both public and private funds, resulting in fragmented healthcare systems. Importantly, the objective of providing effective universal coverage has not been completely achieved in most countries.^18^

Regarding public policies for cancer control, most countries in the region have developed or are going through the implementation phase of National Cancer Control Plans. Uruguay has the most advanced cancer registry in the region, and local and regional important achievements have also been reported in the population- and hospital-based registries in Cali and some provinces in Argentina.^19^

With regard to primary prevention and legislation, the antitobacco act and its enforcement in Uruguay, Chile, and Colombia and the obesity or malnutrition act in Uruguay, Chile, Colombia, and Mexico should be underscored. Vaccination programs for the human papilloma virus and hepatitis have been implemented in most countries.

Regarding early detection, most countries have national screening programs for breast cancer and cervix cancer, and Uruguay and Argentina have those for colon cancer.

## COVID-19 PANDEMIC AND MEASURES IMPLEMENTED BY THE GOVERNMENTS OF THE REGION

The first cases of COVID-19 infection in the region were reported between the last week of February 2020 (eg, February 28 in Brazil) and the beginning of the third week of March 2020 (eg, March 17 in Guatemala). Facing the healthcare emergency, the governments in the region enforced the guidelines drafted by the WHO, mainly social distancing. Some days later, and considering when the first cases were reported, governments implemented the closing of both air and land borders. These lockdown measures were accompanied by either partial or complete suspension of public transportation, which limited social mobility and considerably reduced the flow of patients in healthcare centers.^20^ Social distancing, handwashing, the use of masks, diagnostic tests, and follow-up of cases and contacts were the most important prevention measures implemented.

Facing the pandemic, the healthcare systems in the region allocated both economic and social resources and healthcare infrastructure in response to the prevention, diagnosis, and COVID-19 management challenge.

In collaboration with governments, medical societies and oncology societies implemented the recommendations and guidelines drafted by the WHO and international societies such as ASCO and European Society for Medical Oncology, and in some countries (Argentina, Uruguay, Chile, Brazil, Peru, Colombia, and Honduras), local guidelines were drafted for patient care depending on the characteristics of each country.

Most countries implemented telemedicine programs as an alternative option, that is, phone visits or remote Internet visits.

## REGIONAL IMPACT OF THE PANDEMIC ON CANCER CONTROL

Latin American healthcare systems are characterized by fragmented health systems, weak social protection for disadvantaged people, and significant percentages of the population living in poverty. Therefore, the pandemic will have a greater impact on cancer control in our region compared with countries with better public healthcare systems.

In this study, we compared 2019 and 2020 data from the above-mentioned countries for March 16-June 30 and found a significant reduction—variable depending on the country—in the number of first-time visits to oncology services (between –28% and –38%; Table 1) and a corresponding reduction in pathology (between –6% and –50%; Table 2), surgery (between –28% and –70%; Table 3), and chemotherapy (between –2% and –54%; Table 4) for patients with cancer. The available data on radiotherapy showed a significant reduction in the case of Chile (public subsector sample) and Peru (private provider) (Table 5). On the other hand, and in accordance with international recommendations to postpone screening studies in average-risk patients, a significant reduction in PAP smear test studies (between –46% and –100%), mammography (between –32% and –100%), and fecal occult blood test (Uruguay: –73%) was found (Table 6).

**TABLE 1:**
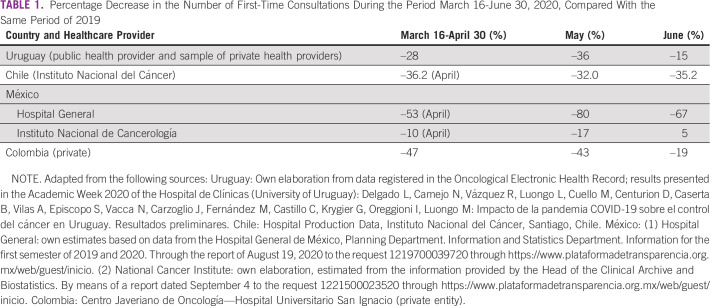
Percentage Decrease in the Number of First-Time Consultations During the Period March 16-June 30, 2020, Compared With the Same Period of 2019

**TABLE 2:**
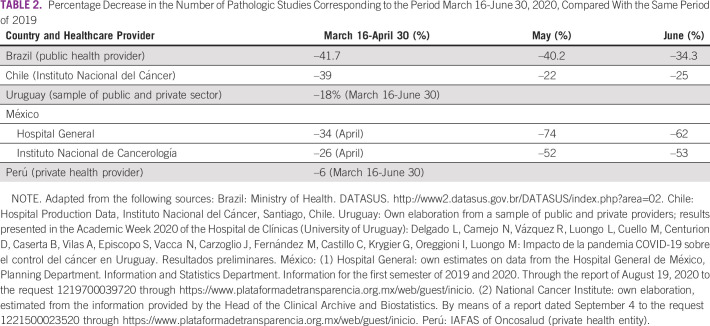
Percentage Decrease in the Number of Pathologic Studies Corresponding to the Period March 16-June 30, 2020, Compared With the Same Period of 2019

**TABLE 3:**
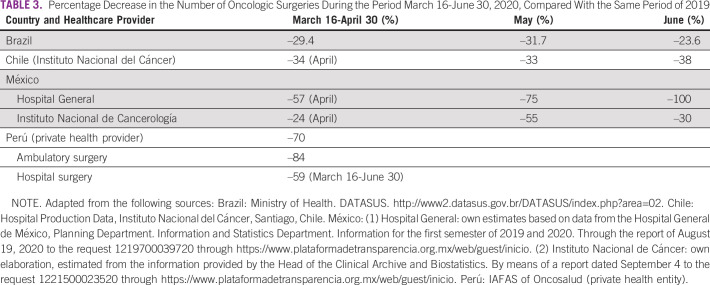
Percentage Decrease in the Number of Oncologic Surgeries During the Period March 16-June 30, 2020, Compared With the Same Period of 2019

**TABLE 4:**
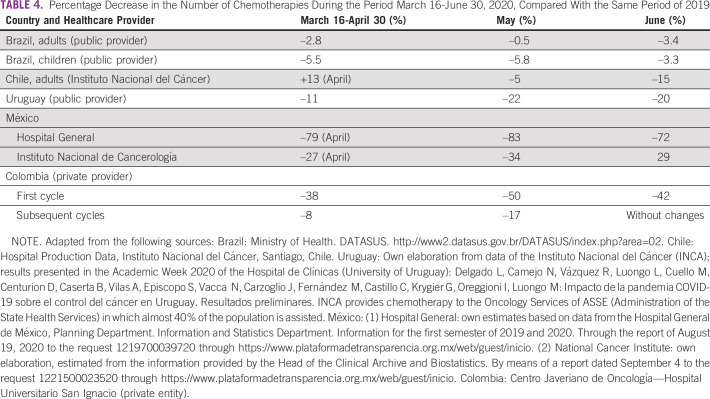
Percentage Decrease in the Number of Chemotherapies During the Period March 16-June 30, 2020, Compared With the Same Period of 2019

**TABLE 5:**
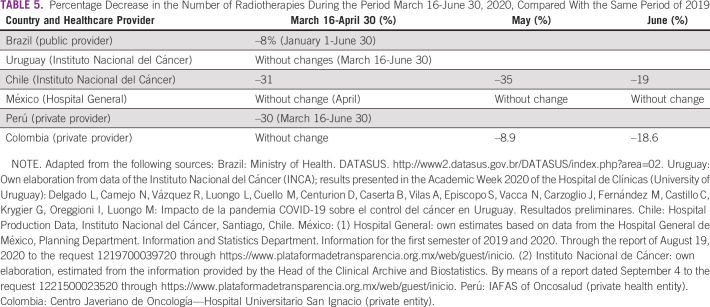
Percentage Decrease in the Number of Radiotherapies During the Period March 16-June 30, 2020, Compared With the Same Period of 2019

**TABLE 6:**
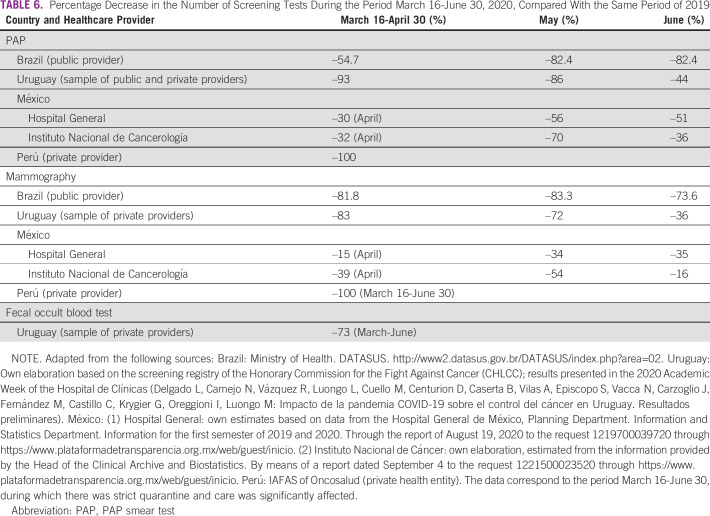
Percentage Decrease in the Number of Screening Tests During the Period March 16-June 30, 2020, Compared With the Same Period of 2019

If this situation becomes a trend, the health and economic impact will be compounded in the postpandemic period, with an overload of demand on health services to ensure diagnostic tests and consequent treatments. This will, in turn, negatively affect the level of resources available for cancer control.

Cancer mortality will increase, both in the short and medium term, as a result of less control of risk factors, delays in prevention screening tests, less access to cancer diagnoses and treatments, and an increase in poverty generated by the pandemic.

Direct expenses associated with cancer control, which are those incurred within the health system, will increase in the medium term, primarily because of late-stage diagnoses, which are associated with higher costs of care.

## COSTS OF CANCER CARE AND POSSIBLE IMPACT OF THE PANDEMIC ON CANCER EXPENSE

Most Latin American countries do not have evaluations on the costs of cancer care, either at the aggregate level or associated with the different cancers and the different stages of the disease, and almost none have information on indirect costs, that is, the resulting costs of productivity and income losses for patients and their families as a result of premature morbidity and mortality. For this report, it was possible to analyze data from Brazil, Peru, Uruguay, and Mexico, which is summarized below.

In Brazil, spending on cancer care has had a continuous expansion in recent years and it is estimated that total spending (direct and indirect costs) will reach 76 billion USD in 2020. A recently published study estimates the average cost of cancer as 1.7% of gross domestic product (GDP) per year, reaching almost 60 million USD in 2015.^21^

In Peru, the Budget Program for Cancer Prevention and Control-024 has significantly increased the allocation of budget resources for cancer prevention and control at the national level.^22^ The budgetary resources assigned to this program for the 2017-2019 triennium increased by 29.49% compared with the period 2011-2016, reaching 588 million USD. In addition, it should be noted that there was a reorientation of the allocations, with an increase of 14% in what was allocated to treatment, which went on to represent 44.4% (260 million USD) for the years 2017-2019.

Uruguay has information on spending on cancer drugs included in universal coverage and granted in legal actions, radiotherapy, pathology, and positron emission tomography-computed tomography. Based on this partial information, direct spending in 2019 amounted to $82 million USD. Considering that health spending reaches approximately 9.5% of GDP in 2019, what is allocated to cancer has a weight of at least 1.63% in the stated spending, a percentage similar to that reported by Brazil.

In Mexico, three public institutions focus on more than 96.78% of the national population: Instituto Mexicano del Seguro Social, Instituto de Salud y Seguridad Social para los Trabajadores del Estado, and the operational part known as Seguro Popular.^23^ Between 3% and 11% of the budget of these institutions is allocated to cancer care. Regarding the costs of studies and treatments for cancer, it was possible to access data referring to breast cancer care with funds from the Seguro Popular de México with a program that operated from 2003 to 2019 (corresponding public information of this program is available in ref. 24). The total annual budget per patient was 135,372 USD on average. When direct costs for systemic treatment are analyzed according to the stage and biological profile of breast cancer (hormone-sensitive, human epidermal growth factor receptor 2 [HER2]–positive, HER2-negative, or triple-negative), it is observed that for HER2-negative breast cancer, the stage IV treatment reaches 6,368 USD per patient, doubling the cost of treating patients with stage IIB-III and tripling that of those with stage IA and IIA. If hormone therapy is associated with an inhibitor of cyclin D1/CDK4 and CDK6, such as ribociclib, the cost per patient would be more than 20 times higher than the cost of treating invasive cancer diagnosed in earlier stages and approximately 600 times the cost of treating noninvasive carcinoma (stage 0). For patients with metastatic HER2-positive breast cancer (23% of breast cancers in Mexico), the cost per patient of treatment with trastuzumab, pertuzumab, and chemotherapy doubles the cost for the treatment of patients with disease in earlier stages (38,796 *v* 21,000 USD) and is 154 times higher than the cost for patients with carcinoma in situ (stage 0), whose average is 251.45 USD.

These results are consistent with those published by European and North American countries^25-27^; however, the level of resources and economic capacity in Latin American countries is much lower, which allows predicting a more deleterious economic impact for our health systems.

## PROPOSED PREVENTION AND MITIGATION MEASURES

To reduce the negative impact of COVID-19 on cancer control at the regional level and avoid generating an uncontrollable situation in the coming years in public health, we propose the following measures that take into account the characteristics of the population and healthcare systems and the level of resources in Latin American countries:

- Guaranteeing access to oncology services, including support for patient transportation, elimination of economic barriers (out-of-pocket payments), and the use of communication technologies for remote patient assessment and monitoring.
- Developing communication and education programs that appropriately guide patients with cancer to understand the risk of infection by SARS-CoV-2 versus the risk of inappropriate control of their disease such as missing treatments for fear of the virus.
- Developing measures that reduce the pandemic's impact on conditions related to poverty, including strategies for reducing the economic impact of cancer on patients through establishment of ongoing dialogue between the health sector and other sectors of the economy. The desired result is balanced measures protective of health and life and protective of the economic infrastructure.
- Enabling various levels of care and nonspecialized professionals to be involved in oncology and the care of patients with cancer, through the appropriate use of communication tools, the constitution of care networks, and structuring of clinical referrals with different levels of responsibility.
- Generating normative operational and economic frameworks that enable and facilitate the implementation of telemedicine.
- Developing strategic operational plans for phased reintroduction of activities for early detection of cancer with the aim of reducing the risk of late diagnoses without overwhelming the capacity of oncology services.
- Adapting clinical practice guidelines for the management of patients with cancer according to local pandemic situations and best available evidence and with adjustments made to accommodate the level of resources and characteristics of the health system.
- Maintaining, or resuming as soon as possible, measures for control of risk factors, particularly tobacco use, the harmful consumption of alcohol, obesity, and sedentary lifestyles and vaccination against human papilloma virus and hepatitis B.
- Fast-tracking regional registry systems to assess the impact of the pandemic on cancer care.
- Promoting the development of research on the intersection of COVID-19 and cancer, including its impact on patients, oncology services, and health personnel.
- Promoting regional collaboration and the exchange of learning among government, academic, and healthcare institutions.

Although the proposed actions should be implemented immediately in response to the pandemic, we consider that they should take place within the framework of the progressive strengthening of health systems, which includes actions such as the following:

- Ensuring the necessary infrastructure for the prevention, early detection, and treatment of cancer.
- Guaranteeing access to and coverage of essential cancer-related services.
- Empowering patients, their families, and civil society groups to advance cancer prevention, diagnosis, treatment, and rehabilitation.
- Developing, promoting, and implementing national cancer control programs in the face of the new public health realities; defining financing mechanisms, which will protect cancer care in the face of new priorities in public health; and developing flexible operational tools in preparation for inevitable and increasing health threats.
- Strengthening the implementation of population-based cancer registries using publicly available information to enable real-time planning, monitoring, and evaluation of cancer control plans and agile adjustment and deployment of cancer prevention and control policies.
- Developing evidence-based clinical practice guidelines stratified according to available resources (human, diagnostic, and therapeutic) to ensure rational use of resources and achieve the best possible care.
- Developing administrative databases to understand aggregated expenses associated with cancer care, by disease stage and site, to promote more efficient use of resources.
- Promoting well-trained teams of biostatisticians and health economists for evaluating the quality of the data collected and for useful analysis.
- Supporting innovation and the development of translational academic research.
- Following WHO's recommendation, creating, in each country, an integrated health system that improves the current inequitable segmentation and fragmentation of health systems in many countries of the region. Additional priorities are universal access to, and health coverage in, health systems that abide by the Social Security principles and UN and The International Labour Organization guidelines.

The coronavirus pandemic provides an opportunity for the society to act in solidarity and find in this crisis the impetus to achieve the Sustainable Development Goals: Goal 3, Health and Well-Being; Goal 10, Reducing Inequalities; and Goal 17, Developing Alliances to accomplish the proposed objectives.

## Appendix. CONTRIBUTORS (sorted by country)

**Mónica Ventriglia—Buenos Aires, Argentina:** MD, Specialist in Medical Oncology, Director of PREAIDEO, Asistencia Integral al Enfermo Oncológico. Clinical Consultant of the Sociedad Latinoamericana y del Caribe de Oncología Médica (SLACOM).

**María Celeste Díaz—Buenos Aires, Argentina:** MD, Specialist in Medical Oncology (UBA). MSc Candidate in Health Economics. Former Coordinator for STDs at National Cancer Institute—Argentina. Technical Consultant for Secretariat of Medication and Strategic Information—Ministry of Health.

**José Gomes Temporão—Río de Janeiro, Brazil:** MD, Researcher FIOCRUZ/RJ, Past-Minister of Health (2007-2010).

**Sandro J. Martins—Brasilia, Brazil:** MD, Specialist in Medical Oncology, Investigator FIOCRUZ/DF Hospital Universitário de Brasília-DF—EBSERH.

**Mario Roberto Dal Poz—Rio de Janeiro, Brazil:** MD, Professor Institute of Social Medicine—University of Estado do Rio de Janeiro (UERJ, WHO's Past-Coordinator of the Human Resources in Health [2002-2012]).

**Walter Zoss—Rio de Janeiro, Brazil:** Journalist, Technical Consultant—FIOCRUZ/RJ, CEO—RINC-SLACOM.

**Alessandra de Sá Earp Siqueira—Rio de Janeiro, Brazil:** MD, Physician at National Cancer Institute-Brazil and Federal University of Rio de Janeiro, Specialist in Project Planning and Management and Health Economics—Fundação Getúlio Vargas.

**Tania Alfaro Morgado—Santiago, Chile:** MD, Master in Public Health, Professor, Public Health School, Universidad de Chile (en lugar de University of Chile).

**Osvaldo Artaza Barrios—Santiago, Chile:** MD, Pediatric Cardiologist, Past-Health Minister, Past WHO-PAHO Consultant, Dean Faculty of Health Sciences, University of the Americas.

**Roberto Estay Miquel—Santiago, Chile:** MD, Specialist in Medical Oncologist and Internal Medicine, Master in Public Health, President of the Department for Health Policies of the Chilean College of Physicians.

**Rafael Urriola—Santiago, Chile:** Health Economist, Master in Public Economics and Planning (Universidad Paris X, Nanterre, Francia). Past-President, Asociación Economía de la Salud—Chile, Coordinator Revista Economía de la Salud—Chile.

**Wilson Cubides Martinez—Bogotá, Colombia:** MD, Master in Health Administration and International Public Health.

**Raul Murillo—Bogotá, Colombia:** MD, Master in Public Health. Director of the Centro Javeriano de Oncología—Hospital Universitario San Ignacio, Pontificia Universidad Javeriana.

**Alejandra Zavala—Tegucigalpa, Honduras:** MD, Specialist in Medical Oncologist, Hospital General San Felipe.

**Lourdes Salazar—Guatemala, Guatemala:** MD, Internist. Specialist in Evaluation of Physical Disability.

**Christian Murray, MSc—Guatemala, Guatemala:** Specialist in Health Economics. Master in Health Economics and Politics. Regional Coordinator of Health Economics of the Centro de Estudios en Salud de la Universidad Del Valle de Guatemala.

**Julio César Zúniga—San Pedro Sula, Honduras:** MPH Candidate, University of Michigan.

**Karla Zepeda—Tegucigalpa, Honduras:** MD, Master in Public Health, Hospital Escuela Tegucigalpa.

**Maria de los Angeles Mendoza—Tegucigalpa, Honduras:** MD, Fundación Hondureña para el Niño con cáncer.

**Pedro Estrada—San Pedro Sula, Honduras:** MD, Specialist in Medical Oncology, Hospital Mario Catarino Rivas San Pedro Sula.

**Rolando Medina Barahona—San Pedro Sula, Honduras:** MD, Especialista en Oncología Médica, Instituto Hondureño de Seguridad Social Regional San Pedro Sula.

**Jean René Clemenceau—Ciudad de México DF, México:** MD, Specialist in Oncology, Hospital Angeles Pedregal, Ciudad de México, México. President of the Latin American School of Oncology (ELO).

**Marisol Torres Toledano—Ecatepec, Estado de México, México:** MD, Internist, Master in Health Services Management. PhD Candidate in Health Economics. Hospital General de Zona con Unidad de Medicina Familiar No 76. México Oriente IMSS.

**Jorge Pérez Romero—Ciudad de México, México:** Master in Public Health. Latin American School of Oncology (ELO).

**Omar Gómez Cruz—Tapachula, Chiapas, México:** MD, Surgical Oncologist. President of Fundación Salud y Bienestar Mesoamérica (FUNSALBARME). Tapachula, Chiapas, México.

**Efren Flores Alvarez—Aguascalientes, México:** MD, Surgical Oncologist. Centenario Hospital Hidalgo.

**Adalberto Flores Coutiño—Tapachula, Chiapas, México:** MD, Gynecologist Oncologist. Centro Estatal de Cancerología.

**René Estrada—Tapachula, Chiapas, México:** Master in Epidemiology. Fundación Salud y Bienestar Mesoamérica (FUNSALBARME).

**Teresa Apresa—Ciudad de México, México:** Specialist in Public Health. Hospital de Oncologia. Centro Médico Nacional SXXI, IMSS.

**Adriana González Delgado—Ciudad de México, México:** MD, PhD in Collective Health, Universidad de la Salud.

**Alfredo Aguilar Cartagena--Lima, Perú:** MD, Medical Oncologist. Master in Public Health, Director Revista Carcinos, Senior Scientific Advisor of Auna-IDEAS Foundation.

**Manuel Villaran Iturri–Lima, Peru:** MD, MPH, MSc, Medical Project Manager, AUNA; Sub-Director of Auna-IDEAS Foundation.

**Elena Tapia López - Lima, Perú:** MD, Master in Science Candidate. Head of Health Technology Assessment and Health Economics, AUNA Perú.

**Rodolfo Vázquez—Montevideo, Uruguay:** MD, Health Services Administration Specialist. Professor in Preventive and Social Medicine. University of Uruguay.

**Alvaro Luongo—Montevideo, Uruguay:** MD, Radiation and Medical Oncologist. Professor of Radiation Oncology, University of Uruguay. Past-Director of the National Cancer Institute-Uruguay. Past-President Honorary Commission for the Fight Against Cancer.

**Miguel Fernández Galeano—Montevideo, Uruguay:** MD, Specialist in Health Services Administration. Past-Vice Minister of Health.

**Ida Oreggioni—Montevideo, Uruguay:** Economist, Health Economics Specialist, Past-Director of the Health Economics Area of the Ministry of Health.
